# Novel Peptide with Specific Calcium-Binding Capacity from *Schizochytrium* sp. Protein Hydrolysates and Calcium Bioavailability in Caco-2 Cells

**DOI:** 10.3390/md15010003

**Published:** 2016-12-27

**Authors:** Xixi Cai, Jiaping Lin, Shaoyun Wang

**Affiliations:** 1 The Key Lab of Analysis and Detection Technology for Food Safety of the MOE, College of Chemistry，Fuzhou University, Fuzhou 350108, China; caixx_0123@163.com; 2 College of Biological Science and Technology, Fuzhou University, Fuzhou 350108, China; kathleen369@163.com

**Keywords:** *Schizochytrium* sp., protein hydrolysate, calcium-binding peptide, structure, bioavailability

## Abstract

Peptide-calcium can probably be a suitable supplement to improve calcium absorption in the human body. In this study, a specific peptide Phe-Tyr (FY) with calcium-binding capacity was purified from *Schizochytrium* sp. protein hydrolysates through gel filtration chromatography and reversed phase HPLC. The calcium-binding capacity of FY reached 128.77 ± 2.57 μg/mg. Results of ultraviolet spectroscopy, fluorescence spectroscopy, and infrared spectroscopy showed that carboxyl groups, amino groups, and amido groups were the major chelating sites. FY-Ca exhibited excellent thermal stability and solubility, which were beneficial to be absorbed and transported in the basic intestinal tract of the human body. Moreover, the calcium bioavailability in Caco-2 cells showed that FY-Ca could enhance calcium uptake efficiency by more than three times when compared with CaCl_2_, and protect calcium ions against dietary inhibitors, such as tannic acid, oxalate, phytate, and Zn^2+^. Our findings further the progress of algae-based peptide-calcium, suggesting that FY-Ca has the potential to be developed as functionally nutraceutical additives.

## 1. Introduction

Marine algae, which have traditionally formed part of the diet for centuries, especially in Asian countries such as China, Korea, and Japan, have become a popular research topic because of their biological implication [1]. *Schizochytrium* sp., belonging to marine fungi, possesses a large number of bioactive substances beneficial to the human body, such as unsaturated fatty acids, pigments, and proteins [2]. *Schizochytrium* sp. has been widely used in the industrial production of docosahexaenoic acid. The remaining by-products, containing about 41% protein, are usually used for biological baits or just discarded as industrial waste. Preparation of bioactive peptides from proteins through enzymatic hydrolysis has been a hot topic [3,4]. Therefore, the utilization of protein from the defatted *Schizochytrium* sp. by-products presents an opportunity.

Calcium is the most abundant mineral in the human body, mostly stored in the bones and supporting their structure and function. Calcium deficiency may result in many diseases, such as osteoporosis, kidney stones, and arterial hypertension [5,6]. Therefore, numerous calcium-fortified medicines and foods have come to market. However, calcium deficiency is still widespread due to insufficient absorption of the intake calcium. Ionized calcium is the primary calcium supplement for humans, but intestinal absorption of ionized calcium could be easily affected by the presence of dietary factors, such as tannin, phytate, oxalate, and other divalent metal ions [7]. Thus, a new class of calcium-enriched nutrients that can overcome these shortcomings has the potential to improve calcium nutrition. Organic calcium supplements show their superiority. Calcium-binding peptides, one of the organic calcium supplements, such as casein ophosphopeptides (CPPs) [8], soybean protein hydrolysates [9], whey protein hydrolysates [10], and serum protein hydrolysates [11], have been reported to be capable of promoting calcium uptake. Among these, CPPs were known as excellent mineral carriers with a significant role in promoting calcium ion absorption through the formation of CPP-Ca aggregates and maintaining the solubility [8,12]. CPPs induced calcium uptake in Caco-2 cells involved the transient receptor potential cation of the vanilloid subfamily V member 6, TRPV6 channel, also designated as calcium transporter-1, or CaT1 [13]. In the previous study, the nanocomposites of *Schizochytrium* sp. protein hydrolysate (SPH) chelated with calcium ions were prepared and the characterization of nano-composites was investigated by our group [14]. However, none has been reported about purified peptide with specific calcium-binding capacity from *Schizochytrium* sp. protein hydrolysate and calcium bioavailability. The research on the purified peptide is necessary to further understand the relationship between structure and function, and action mechanism.

The objectives of this study were, therefore, to isolate and characterize specific calcium-binding peptides from *Schizochytrium* sp. protein hydrolysate (SPH) and explore the possible chelating mechanism. Additionally, the Caco-2 cell monolayer model was used to determine the calcium bioavailability. This study could provide a new train of thought of the calcium-binding peptide from *Schizochytrium* sp. protein hydrolysate for the potential to be developed as a new kind of functionally nutraceutical supplements to improve bone health in the human body.

## 2. Results and Discussion

### 2.1. Purification of Calcium-Binding Peptide

*Schizochytrium* sp. protein hydrolysates consisted of various peptides were confirmed to possess calcium-binding capacity [14]. Systematic investigation on the calcium-binding properties of various peptides in SPH is of great importance. For this purpose, a specific peptide with calcium-binding capacity was first purified.

**Figure 1:**
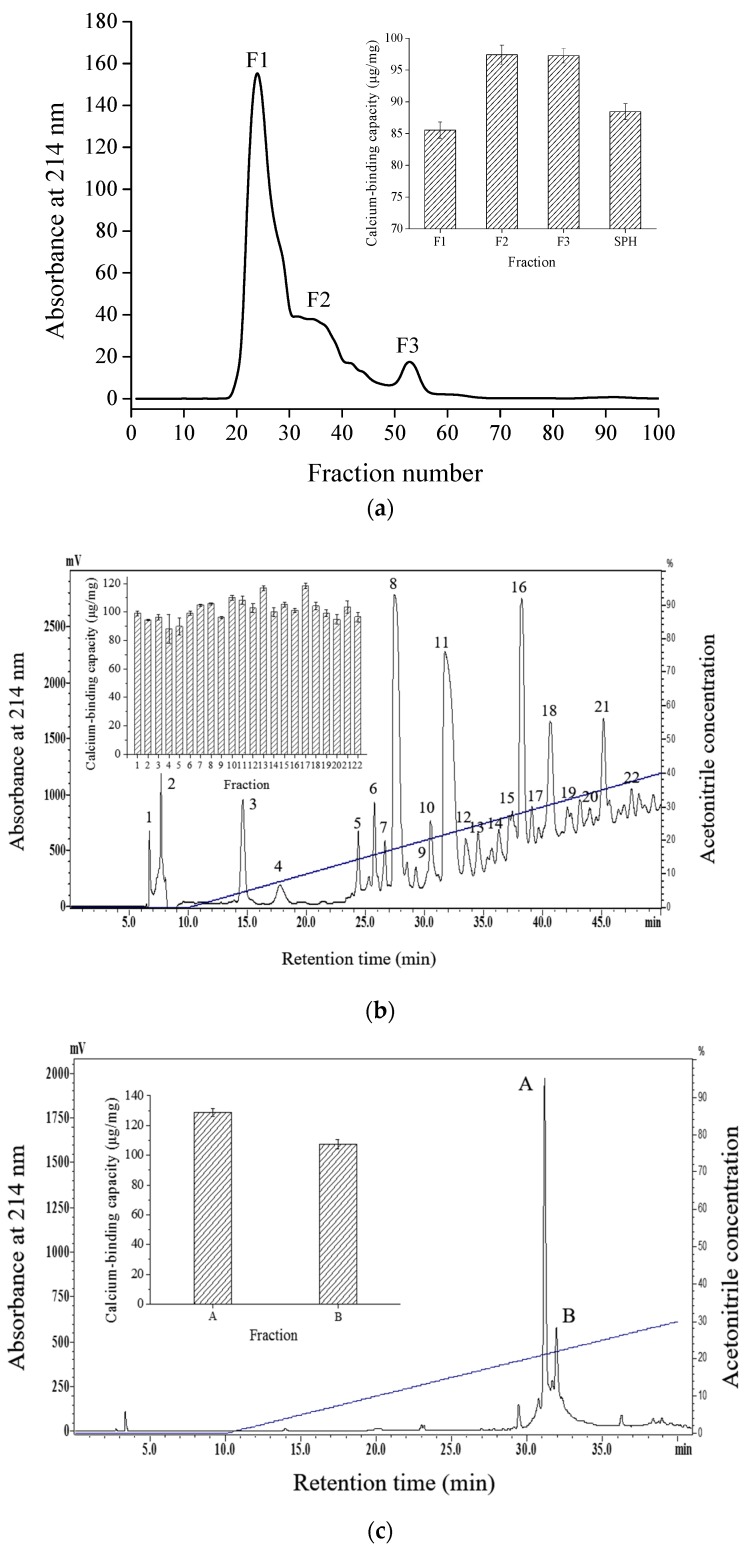
Elution profiles and calcium-binding capacities of calcium-binding peptides. (**a**) Sephadex G-25 gel filtration chromatography of SPH; (**b**) semi-preparative C18 RP-HPLC of fraction F3; and (**c**) analytic RP-HPLC of fraction 17 from semi-preparative HPLC.

As shown in Figure 1a, SPH was divided into three size-dependent fraction through Sephadex G-25 chromatography. The calcium-binding capacities of F2 and F3 were similar and remarkably higher than F1 and SPH. Many studies have shown that peptides with lower molecular mass exhibited higher chelating capacity [15,16,17]. Therefore, the active fraction F3 with lower molecular mass was pooled and loaded onto semi-preparative C18 RP-HPLC. Twenty-two distinct fractions were collected and all of them exhibited different degrees of calcium-binding capacities (Figure 1b). Among them, activities of fraction 13 and fraction 17 were significantly higher than other fractions and fraction F3 from Sephadex G-25 chromatography (*p* < 0.05). Fraction 17, which showed the highest chelating capacity, was first selected for further purification by analytic RP-HPLC. Finally, fraction A, with the highest calcium-binding activity (128.77 ± 2.57 μg/mg), was collected and lyophilized for further studies (Figure 1c).

### 2.2. Identification of the Calcium-Binding Peptide

**Figure 2:**
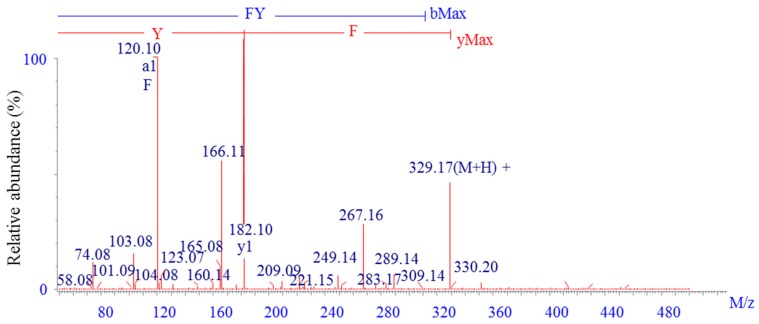
Identification of the amino acid sequence of the calcium-binding peptide using LC-ESI-MS/MS.

The amino acid sequence of fraction A was determined to be Phe-Tyr (FY) with a molecular weight (MW) of 328.17 Da using liquid chromatography-electrospray ionization-tandem mass spectrometry (LC-ESI-MS/MS) (Figure 2). Subsequently, the identified peptide was chemically synthesized and the calcium-binding capacity was determined to be 125.91 ± 1.63 μg/mg, which was equivalent to the purified sample. Calcium-binding peptides from various sources with different MW and sequences have been isolated. Jeon reported that a peptide purified from *Chlorella* protein hydrolysates had a calcium binding activity of 0.166 mM and was determined to be 700.48 Da [18]. In our previous works, four dipeptides or tripeptides from whey protein hydrolysates were confirmed to possess 70–80 μg/mg calcium-binding capacity [19,20,21,22]. Not only the differences in the length and net charge of peptides, but also the different amino acid composition and sequence, could affect the extent of chelate formation with divalent metal cations [15,23]. Previous reports showed that the phosphorylation of tyrosine residues could provide appropriate chelating sites for positively charged metals, like calcium, zinc, and iron [24]. Kim indicated that an iron-binding peptide separated from heated whey hydrolysates contained 16.58% of phenylalanine residues, which was higher than other amino acids [25]. Moreover, dipeptide or tripeptide was deemed to promote metal ion absorption more effectively than higher MW peptides in intestinal epithelial cells [26]. Consequently, both of the Phe and Tyr residues in the purified peptide might contributed to chelation with metal cations.

### 2.3. Structural Characterization of Peptide-Calcium Chelate

#### 2.3.1. Ultraviolet Spectroscopy Analysis

**Figure 3:**
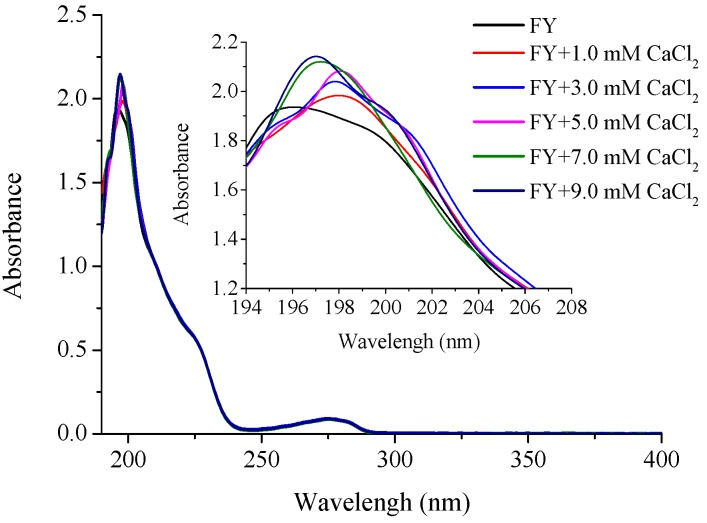
UV spectra of FY with different CaCl_2_ concentrations over the wavelength range from 190 to 400 nm.

Aromatic amino acids including tryptophan, phenylalanine, and tryptophan residues, could produce different UV spectra because of different chromophores. Phenylalanine has a specific absorption peak at 260 nm, and tyrosine at 280 nm approximately [27]. Therefore, the UV spectra was utilized to discuss the chelating mechanism of FY. As shown in Figure 3, the UV absorption spectra of FY-Ca chelates presented distinct differences from that of FY, which implied that a new substance was formed when FY interacted with calcium ions. Dipeptide FY had a maximum UV absorption peak at about 196 nm. With the increase of calcium ion concentration, the absorbance of the maximum absorption peak gradually increased from 1.937 to 2.149, showing a hyperchromic effect and redshift phenomenon. The results indicated that the chromophore groups (-C=O, -COOH) and auxochrome groups (-OH, -NH_2_) generated polarizing changes when the ligands bound with calcium ions in the chelating process [28,29]. In addition, both FY and FY-Ca chelate had specific absorption peaks near 280 nm with the same intensity, suggesting that the tyrosine structure remained unchanged and the phenolic hydroxyl group of Tyr in FY was not involved in the chelation reaction because of the steric hindrance of the benzene ring. Hence, it could be presumed that the nitrogen atom of -NH- and -NH_2_ and the oxygen atom of -C=O and -COOH participated in the chelation.

#### 2.3.2. Fluorescence Spectroscopy Analysis

**Figure 4:**
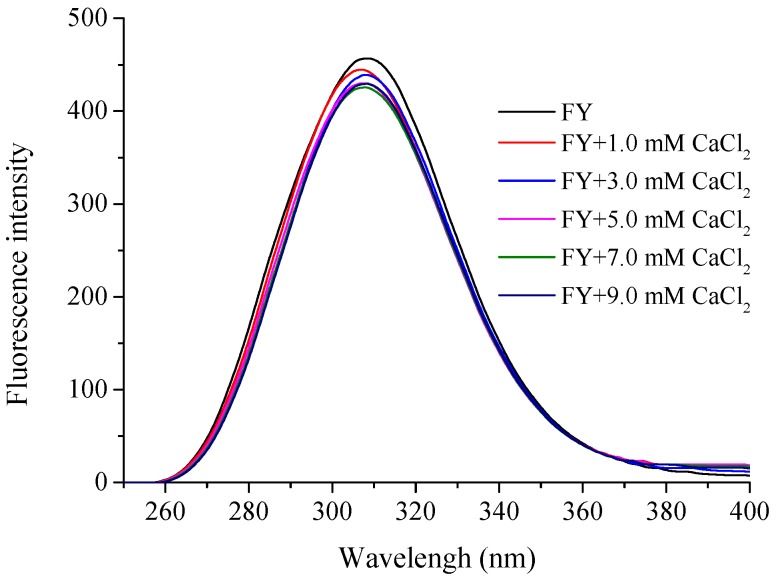
Fluorescence spectra of FY with different CaCl_2_ concentration over the wavelength range from 295 to 500 nm.

The specific calcium-binding peptide FY included Phe and Tyr, which could generate endogenous fluorescence at an excitation wavelength of 280 nm, and the corresponding emission peaks of Phe and Tyr were 303 nm and 313 nm, respectively. The fluorescence spectra of FY and FY-Ca chelate were shown in Figure 4. With the increase of calcium ion concentration, the intensity of endogenous fluorescence at 310 nm was reduced, which implied that calcium ions could be chelated by aromatic amino acids and lead to fluorescence quenching. Particularly, obvious endogenous fluorescence quenching appeared as soon as 1.0 mM of CaCl_2_ was introduced. However, when the concentration of calcium ion reached 5.0 mM, no further changes were observed. This potentially manifested that changes in the fluorescence occurred when calcium chelated with the peptides and excess free calcium made no difference. Similar results has been reported by Zhou that fluorescence quenching was observed when calcium ions combined with the calcium-chelating peptide [30]. Moreover, Wu proved that reduced fluorescence intensity was a classic indicator of peptide folding when ferrous ions chelated with sturgeon protein peptide, and ferrous ions closed to tryptophan residues in the folding process [31]. Therefore, the results demonstrated that the calcium ions chelated with FY might cause folding of the peptide and form a compact structure, which contributed to the decrease in fluorescence intensity.

#### 2.3.3. Fourier Transform Infrared Spectroscopy (FTIR) Measurement

**Figure 5:**
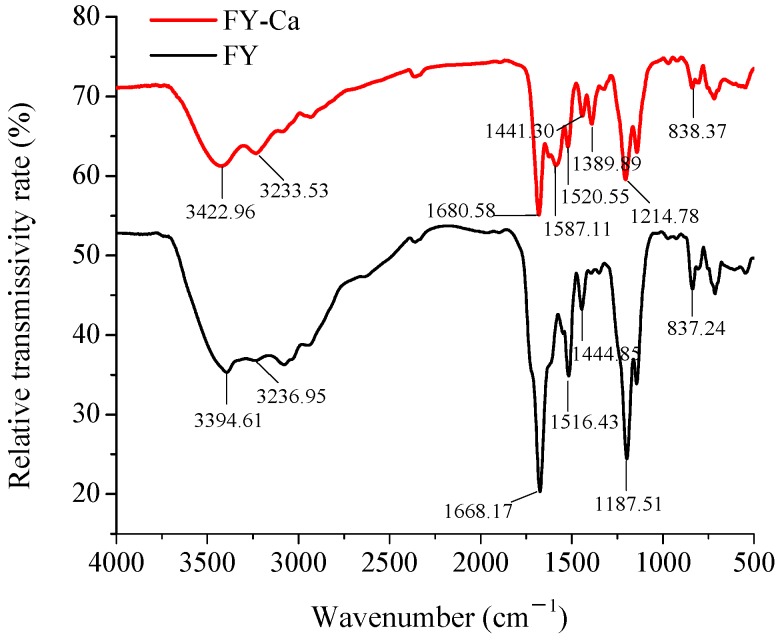
Fourier transform infrared (FTIR) spectra of FY and FY-Ca chelate in the regions from 4000 to 400 cm^−1^.

The specific FTIR absorption peak changes of the amides and carboxylates in FY could reflect the interaction of calcium ions and organic ligand groups of the peptides. As shown in Figure 5, displacement and intensity changes of main absorption peaks could be observed when calcium ions bound with the amino acid residues. The two most important vibrational modes of amides are the amide-I vibration and amide-II vibration, the amide-I vibration is primarily caused by the stretching of C=O bonds, amide-II vibration is assigned to deformation of N-H bonds and stretching of C–N bonds [21,32]. The absorption band of FY at 1668.17 cm^−1^ for the amide I band shifted to a higher frequency (1680.58 cm^−1^) after chelating with calcium, manifesting that the -COO- group participated in the covalent combining reaction with the metal cations [33]. Additionally, the amide II band at 1516.43 cm^−1^ in FY also shifted to 1587.11 cm^−1^ in the FY-Ca chelate. The characteristic peak of amide-A stretching vibration of FY shifted from 3394.61 cm^−1^ to 3422.96 cm^−1^ might be due to the replacement of N-OH bonds (hydrogen bonds) with Ca-N bonds after calcium chelation [22]. After chelation, the spectrum shifted towards high-frequency wavenumbers (3500–2800 cm^−1^), indicating that dipole field effect or induced effect led to the electron cloud density and frequency increased [14]. In the fingerprint region, the absorption intensity at 1187.51 cm^−1^ decreased and moved towards 1214.78 cm^−1^ simultaneously when FY chelated with calcium. A reasonable explanation was that FY bound with calcium ion and form C–O–Ca [14]. Furthermore, the absorption intensity observably reduced at lower frequency 837.24 cm^−1^ in FY-Ca chelate, it might attributed to the changes of -C–H group and -N–H group of FY in the chelating procedure. Previous research showed that the carboxyl group loss of protons and negative electricity (-COO-) was also potential binding site. Additionally, the amino group (-NH_2_) and imino group of the peptide bond (-NH) were also likely to be involved in the formation of chelate [34]. The results of FTIR proved that oxygen atoms of the carboxyl group and nitrogen atoms of the amino group might be involved in the chelating reaction and generated a new substance.

### 2.4. Thermal and pH Stability Analysis of Peptide-Calcium Chelate

#### 2.4.1. Thermogravimetry-Differential Scanning Calorimetry (TG-DCS) Analysis

**Figure 6:**
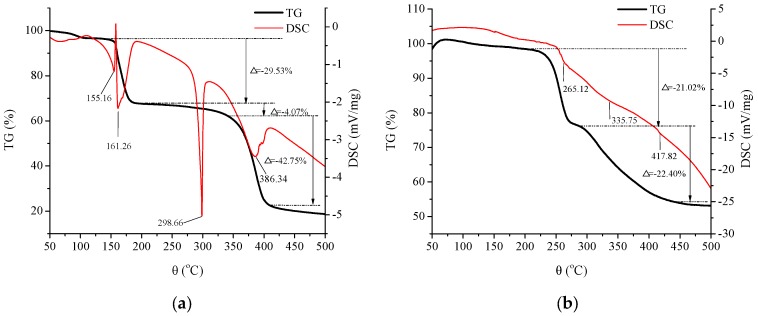
Typical TG-DSC thermograms of (**a**) FY and (**b**) FY-Ca chelate.

The difference of thermostability between FY and FY-Ca chelate was explored through TG-DSC analysis. As shown in Figure 6a, the TG curve of dipeptide FY revealed that the thermal decomposition reaction of FY involved three stages in the whole process of 76.35% weight loss, and the thermal transition temperature was 155.16 °C, 161.26 °C, 298.66 °C, and 386.34 °C, respectively according to DSC analysis. The endothermic peaks were mainly caused by the destruction of C–N bonds in different positions of FY [20]. However, the TG curve of FY-Ca performed only two stages and lost 43.42% of its weight entirely (Figure 6b). The temperature of endothermic peaks significantly shifted to 265.12 °C, 335.75 °C, and 417.82 °C after the calcium ion chelated with FY, suggesting that FY-Ca chelate was less sensitive to thermal denaturation and performed better thermostability than FY, which was advantageous for application in medicine and functional food.

#### 2.4.2. Calcium-Releasing Percentage Assay

**Figure 7:**
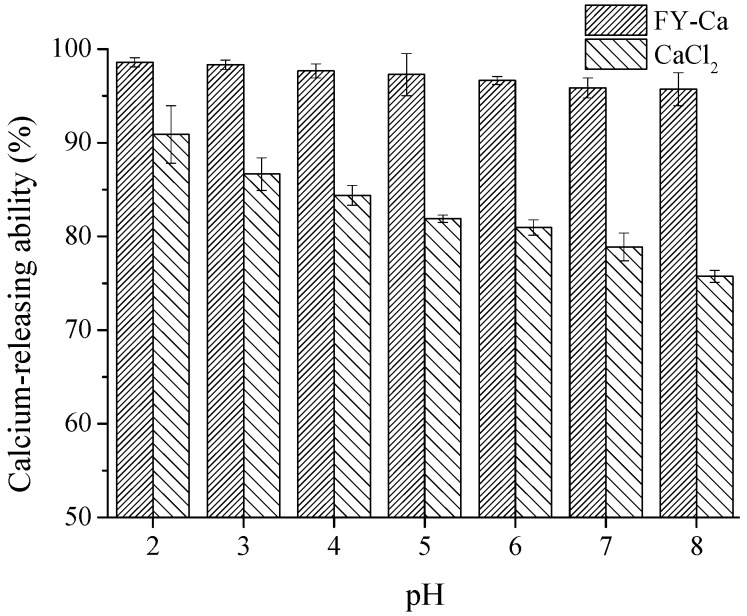
Calcium-releasing percentage of FY-Ca chelate and CaCl_2_ at different pH.

The calcium-releasing percentages of the FY-Ca chelate and CaCl_2_ at different pH were shown in Figure 7. The solubility of FY-Ca and CaCl_2_ was obviously different. The calcium-releasing percentage of CaCl_2_ exhibited a distinctly downward trend with the increase of pH value, and was reduced to 75.7% at pH 8.0, which could deduce that the free calcium ions and OH^−^ formed precipitates and led to a decline in the percentage. In contrast, the calcium-releasing percentage of FY-Ca chelate was always apparently higher than that of CaCl_2_ at pH 2.0–8.0, and it maintained a relatively stable value of about 95% as well. The pH value in human intestinal tract is approximately pH 7.2, and FY-Ca chelate had higher solubility and better bioavailability in the gastrointestinal tract, which implied that FY-Ca chelate could be effectively absorbed and transported by intestinal epithelial cells than CaCl_2_ [35].

### 2.5. Calcium Bioavailability in Human Intestinal Caco-2 Cell Lines 

#### 2.5.1. Cell Uptake of FY-Ca

**Figure 8:**
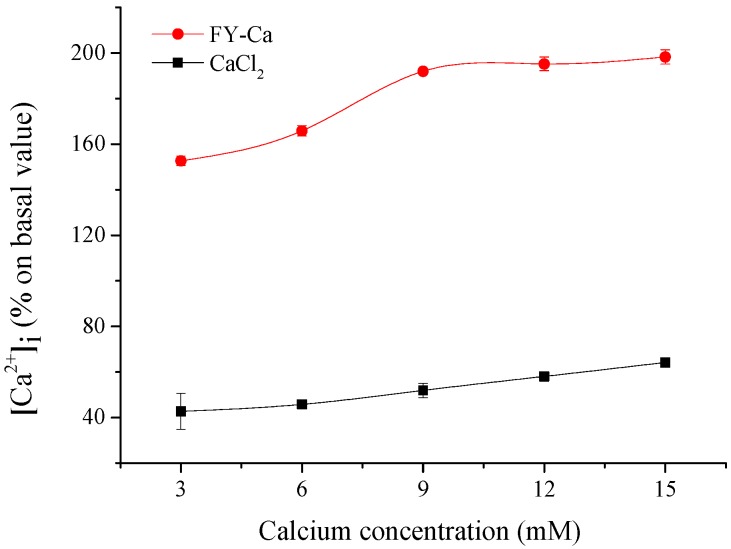
Cell uptake of FY-Ca chelate and CaCl_2_ in Caco-2 cell by Fluo-3-AM loading for fluorescence analysis.

For the uptake studies, Caco-2 cells were pre-incubated with FY-Ca chelate at different concentrations with CaCl_2_ used as control. The effect of FY-Ca on the intracellular calcium concentration increased dose-dependently and then approximately trended to stable when the calcium concentration reached 9 mM, according to results in Figure 8. Additionally, the absorption-enhanced effects of FY-Ca were more than three times that of CaCl_2_ at the same calcium concentration. Similar findings were also reported for desalted duck egg white peptides [36], soybean protein hydrolysates-calcium complex [9], and CPPs [37], which might act as calcium carriers and interact with the plasma membrane to transport calcium to the cytosol and ultimately significantly promote calcium uptake.

#### 2.5.2. Calcium Bioavailability under the Action of Dietary Inhibition Factors

**Figure 9:**
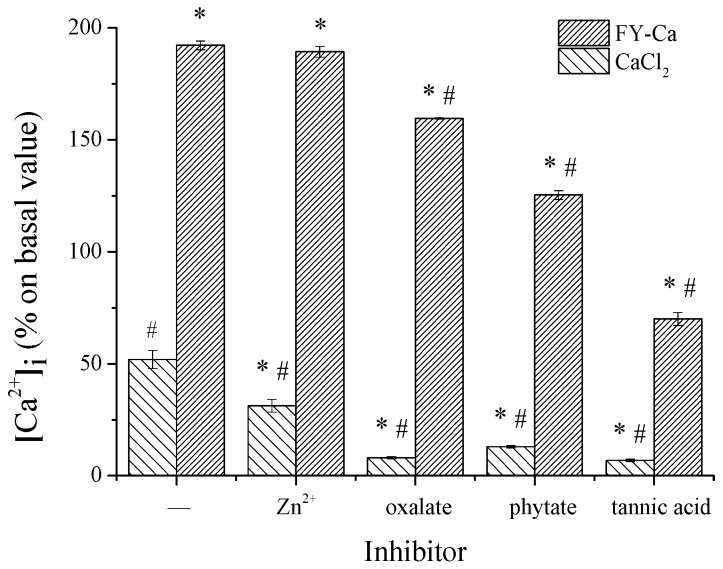
Effect of FY-Ca chelate on calcium bioavailability under the action of dietary inhibition factors. The concentration of calcium was 10 mM and tannic acid/Ca, oxalate/Ca, phytate/Ca, or Zn/Ca = 20:1. * Statistical significance *p* < 0.05, compared with the CaCl_2_ control group. ^#^ Statistical significance *p* < 0.05, compared with the FY-Ca control group.

Well-established dietary factors, such as tannic acid, oxalate, phytate, and zinc ions, were chosen to evaluate whether the typical inhibitors from food would affect the uptake of calcium chelated by FY, with CaCl_2_ as control. As expected, the addition of zinc ions, oxalate, phytate, and tannic acid severely decreased the calcium uptake efficiency of CaCl_2_ by 39.7%, 84.4%, 74.9%, and 86.6%. FY-Ca, by contrast, could protect calcium ions from precipitation caused by oxalate, phytate, and tannic acid, and retain 83.0%, 65.2%, and 36.5% of calcium uptake efficiency, which were 5.3, 2.6, and 2.7 times higher than CaCl_2_, respectively (Figure 9). Furthermore, the addition of Zn ions had little impact on the calcium uptake efficiency of FY-Ca.

Divalent metal ions, such as zinc and ferrous ions, have negative interactions with calcium nutrients and inhibit their uptake since the common receptors for these metal ions, DMT1, are located in the intestine [38]. In this study, the addition of FY could significantly attenuate the inhibition effect of zinc ions on calcium uptake, indicating that FY-Ca might pass through the cell membrane through specific pathways other than the DMT1 receptor. Organic phosphates, such as oxalate and phytate, greatly inhibit calcium uptake due to the formation of insoluble and indigestible complexes [7,39]. In the present study, the calcium uptake efficiency of FY-Ca was superior to CaCl_2_ in the same condition, obviously, which might be due to the stronger chelating power of FY than organic phosphate and prevention of calcium precipitation. Tannin is another dietary factor belonging to polyphenols that exhibits extremely strong protein degeneration and metal ions complexing actions [40]. The addition of tannic acid also decreased the absorptivity of FY-Ca in Caco-2, which might be attributed to the peptide denaturation under high-dose tannic acid. Despite all of these, the calcium uptake efficiency of FY-Ca was remarkably higher than CaCl_2_. These results demonstrated that FY could prevent a great amount of calcium from being precipitated by certain substances, thus improving calcium uptake. The present study provides powerful evidence for the idea that some proteins/peptides could be considered as mineral carriers because of their ability to bind and solubilize calcium with the possible role in increasing calcium transport across intestinal epithelial cells [41].

## 3. Materials and Methods

### 3.1. Materials

The defatted *Schizochytrium* sp. was kindly provided by Fisheries Research Institute of Fujian, China. The commercial protease, Alcalase (EC. 3.4.21.62, 2.2 × 10^5^ U/g) and Flavourzyme (EC. 3.4.11.1, 7.8 × 10^4^ U/g) were products of Novozymes (Copenhagen, Denmark). Sephadex G-25 was purchased from Amersham Pharmacia Co. (Uppsala, Sweden). Methanol and acetonitrile used in liquid chromatography were of HPLC grade. All of the other chemicals and solvents were of analytical grade and commercially available.

### 3.2. Preparation of Schizochytrium *sp*. Protein Hydrolysates

*Schizochytrium* sp. protein was prepared from *Schizochytrium* sp. by alkali extraction and acid precipitation, and *Schizochytrium* sp. protein hydrolysate possessing high calcium-binding capacity was prepared through stepwise enzymatic hydrolysis with Alcalase and Flavourzyme, as described in our previous work [14].

### 3.3. Purification of Specific Calcium-Binding Peptides

The lyophilized SPH dissolved in deionized water was loaded onto a Sephadex G-25 column (100 × 2.0 cm) and then eluted with deionized water at a flow rate of 0.3 mL/min. The absorbance of the elution was monitored at 214 nm and the calcium-binding capacity of the fractions was determined. The fraction with the highest calcium-binding activity from Sephadex G-25 chromatography was pooled and further purified by semi-preparation reversed phase HPLC on a C18 reversed-silica gel chromatograph (Gemini 5 μ C18, 250 × 10 mm; Phenomenex Inc.; Torrance, CA, USA). Elution was performed with solution A (0.05% trifluoroacetic acid (TFA) in water) and solution B (0.05% TFA in acetonitrile) with a gradient of 0%–40% B at a flow rate of 2 mL/min for 50 min. The elution was monitored at 214 nm, and the fractions were collected for calcium-binding capacity analysis. The most active fraction was further purified by analytic HPLC. Buffers A and B were the same as those used in semi-preparative RP-HPLC. Runs were conducted with a liner gradient of 0%–30% solvent B at a flow rate of 1 mL/min.

### 3.4. Identification of Purified Calcium-Binding Peptide

The molecular mass and amino acid sequence of the purified calcium-binding peptide were determined using LC-ESI-MS/MS (Delta Prep 4000, Waters Co., Milford, MA, USA) over the *m*/*z* range of 300–3000.

### 3.5. Synthesis of the Purified Peptide

The purified peptide (Phe-Tyr, FY) was synthesized by GL Biochem Corporation. Ltd. (Shanghai, China) through a solid-phase procedure. The purity of the synthesized peptide was 99.22% by HPLC analysis and the structure of peptide was confirmed by mass spectrometry analysis.

### 3.6. Analysis of Calcium-Binding Capacity

The calcium-binding capacity was measured with ortho-cresolphthalein complexone reagent according to the method described by Wang [35] with some modifications. One milliliter of 9 mM CaCl_2_ was mixed with 2 mL of 0.2 M sodium phosphate buffer (pH 8.0), and then 1 mL of 1 mg/mL of peptides was added to create a competitive environment. The mixture was stirred at 37 °C for 2 h. Afterward, the insoluble calcium phosphate salts was removed by centrifugation at 10,000 rpm for 10 min and the calcium contents in the supernatant were determined by the absorbance at 570 nm after introducing the working solution to the samples.

### 3.7. Structural Characterization of Peptide-Calcium Chelate

#### 3.7.1. Fabrication of Peptide-Calcium Chelate

One-hundred milligrams of lyophilized peptide was dissolved in 10 mL of distilled water, and CaCl_2_ solution was introduced subsequently to a 3:1 ratio of peptide to calcium (*w*/*w*) at pH 6.0. The reaction solution was placed in a shaking water bath at 140 rpm and 37 °C for 20 min. Peptide-calcium chelate was precipitated after introducing absolute ethanol and collected by centrifugation at 10,000 rpm for 20 min.

#### 3.7.2. Ultraviolet Spectroscopy

The ultraviolet spectra of calcium-binding peptide and its calcium chelate were monitored over the wavelength range from 190 nm to 400 nm using an ultraviolet spectrophotometer (UV-2600, UNICO Instrument Co. Ltd., Shanghai, China) as the method described in our previous work with some modifications [14]. For determinations, 20 μg/mL of peptide solution was prepared and the pH was adjusted to 6.5. Then 0, 0.5, 1.0, 1.0, 1.0, and 1.0 μL of 2 M CaCl_2_ was constantly introduced every 10 min and the UV spectra were recorded.

#### 3.7.3. Fluorescence Spectroscopy

Fluorescence spectroscopy was utilized to investigate the conformational changes of the peptide chelating with calcium ions by a Hitachi F-4600 fluorescence spectrophotometer (Hitachi Co., Tokyo, Japan). The excitation wavelength was 285 nm and the emission wavelengths between 250 and 400 nm were recorded. The slit width of excitation and emission was 20 and 30 nm respectively, and the sensitivity was 1. The preparation of sample was the same as that of ultraviolet spectroscopy analysis.

#### 3.7.4. FTIR

One milligram of lyophilized sample and 100 mg of dried KBr were fully mixed and ground in an agate mortar. After tableting, FTIR spectra were recorded at room temperature by an infrared spectrophotometer (360 Intelligent, Thermo Nicolet Co., Madison, WI, USA) from 4000 to 400 cm^−1^. For each spectrum, 64 scans were acquired at 4 cm^−1^ resolution. The peak signals in the spectra were analyzed using OMNIC 8.2 software (Thermo Nicolet Co., Madison, WI, USA).

### 3.8. Thermal and pH Stability Analysis of Peptide-Calcium Chelate

#### 3.8.1. TG-DCS Analysis

A TG-DSC simultaneous thermal analyzer (STA449C, NETZSCH, Bavaria, Germany) was used to analyze the thermostability of the samples. The lyophilized powder samples (5 mg) were set in hermetic pans and heated from 30 °C to 500 °C with programmed heating rate of 10 °C/min and argon flow rate of 30 mL/min.

#### 3.8.2. Calcium Releasing Assay

The calcium ions releasing percentages of peptide-calcium chelate and CaCl_2_ (50 μg/mL in deionized water) were determined at pH ranges of 2.0–8.0. After incubation in a water bath shaking at 140 rpm and 37 °C for 2 h, the reaction solutions were centrifuged at 10,000 rpm for 10 min. The calcium content of the supernatant and the total calcium in the solution were measured using a colorimetric method with ortho-cresolphthalein complexone reagent. The calcium-releasing percentage was calculated as follows:

(1) $$\mathrm{Calcium}\;\mathrm{releasing}\;(\%)=\frac{\mathrm{Calcium}\;\mathrm{in}\;\mathrm{supernatant}}{\mathrm{Total}\;\mathrm{calcium}\;\mathrm{in}\;\mathrm{solution}\;}\times 100$$

### 3.9. The Effect of Peptide-Calcium Chelate on the Cellular Uptake of Calcium

#### 3.9.1. Cell Culture

The human colon adenocarcinoma cells, Caco-2, were grown in dulbecco’s modified eagle medium (DMEM) supplemented with 15% (*v*/*v*) fetal bovine serum (FBS), 1% non-essential amino acid, 100 units/mL penicillin, and 100 μg/mL streptomycin and maintained at 37 °C in a humidified atmosphere with 5% CO_2_. At 80%–90% confluence, cells were seeded on 12-well plastic cell culture clusters at a density of 1 × 10^4^ cells/cm^2^ for seven days.

#### 3.9.2. Fluorescence Analysis for Calcium Bioavailability

Caco-2 cells were pre-incubated with peptide-calcium chelate, CaCl_2_, at different concentrations, and tannic acid/phytate/oxalate/Zn^2+^ plus chelate, and tannic acid/phytate/oxalate/Zn^2+^ plus CaCl_2_, respectively, for 1 h after cells were grown in 12-well plastic cell culture clusters for seven days. The cells were then washed with Hank's balanced salt solution (HBSS, without calcium and magnesium) three times followed by treatment with 10 μM Fluo-3-AM. After incubation for 1 h, cells were washed with HBSS and harvested for analysis by a F-4600 FL spectrophotometer. Intracellular calcium concentrations [Ca^2+^]_i_ are expressed as an increase in fluorescence intensity compared to the baseline, which is the original fluorescence intensity without the addition of exogenous calcium.

### 3.10. Statistical Analyses

All data were presented as means ± standard deviations (SDs) in three replicates. Statistical analysis was performed adopting SPSS 17.0 (SPSS, Chicago, IL, USA). Analysis of variance (ANOVA) was done to determine the significance of the main effects. A confidence level of *p* < 0.05 was considered statistically significant.

## 4. Conclusions

In summary, a specific dipeptide Phe-Tyr (FY) with strong calcium-chelating capacity from *Schizochytrium* sp. protein hydrolysates was purified and the chelating mechanism was investigated. It showed that calcium ions could form dative bonds with carboxyl oxygen atoms and amino nitrogen atoms, as well as nitrogen and oxygen atoms of amido bonds, inducing conformational changes of the dipeptide, and ultimately a new and stable peptide-calcium chelate was formed. The calcium bioavailability of FY-Ca was superior to CaCl_2_, suggesting the potential of FY-Ca to be used as functionally nutraceutical additives.
